# Editorial: PROTACs: Targeted therapies for cancer treatment

**DOI:** 10.3389/fcell.2023.1131638

**Published:** 2023-02-03

**Authors:** Sobia Tabassum

**Affiliations:** Department of Biological Sciences, International Islamic University, Islamabad, Pakistan

**Keywords:** PROTACs (proteolysis targeting chimeras), cancer therapy, targeted drug, technolody development, technology innovation

## Introduction

Cancer is one of the leading cause of morbidity and mortality worldwide. There are several therapies; however, most of them harm both healthy and cancerous cells. Proteolysis-targeting chimaeras (PROTACs) can be utilized as a targeted cancer treatment. These small molecules known as PROTACs are specialized, adaptable, and biologically active. Since PROTACs present a promising and alluring technology to solve off target and drug resistance problems in most cancers. They have the ability to trigger selective intracellular proteolysis, which can be utilized to specifically target and destroy tumor-promoting proteins inside of cells. To enhance their therapeutic outcomes, new understandings of the molecular mechanisms of PROTAC-mediated degradation are required.

## Aim and objectives

The current Research Topic aims to explore the most recent developments in the creation and use of PROTACS-based treatments for cancer. We invite research articles, review articles, and viewpoints that speak to the following issues, but are not restricted to them.

• Molecular underpinnings of PROTAC-mediated protein degradation
• Modulation of the ubiquitination system for cancer treatment
• Innovative designing and development of PROTACS-based therapeutics
• Comparison of PROTACs with earlier non-targeted therapies

This Research Topic brings together several scientific contributions that highlight some extremely intriguing findings under its purview. The published articles on this topic have attracted significant interest from both academia and industry.

Zheng et al. (2022) submitted a comprehensive review on this subject in order to address cancer drug resistance, which is a significant obstacle to the successful treatment of malignancies going forward. Recent developments in this field of study show that PROTACs can successfully destroy mutant targets that confer cancer resistance to first-line therapies, building the foundation for next-generation therapies and providing patients with previously incurable cancers with new chances for remission.

The authors provided an overview of the current use of PROTACs to treat solid tumors and included recommendations for overcoming their clinical development roadblocks. Among designing and development constraints, improvements in bioavailability and safety brought about by an improved delivery route seem to be relevant. In light of this, Juan et al. have proposed methods to enhance their therapeutic efficacy and the development of fine-tuned nanoparticles based delivery systems to increase the bioactivity of PROTACs.

Significant developments in newly developed degrader technologies were also reported in this area (Luo et al.). The emphasis of the authors has been on the molecular design of PROTACs using various methodologies, which provides a deeper mechanistic knowledge of developing degraders and serves as a beneficial roadmap for the advancement of the upcoming degrader technologies. Anwar et al. published a very thorough analysis of PROTACs designed to combat different forms of leukaemia and blood cancers. Along with all of this material, another investigation focused on how YAP1 deacetylation promoted chemotherapy resistance and targeted treatment in FLT3-ITD + AML cells (Meng et al.). This research demonstrates that the FLT3-ITD + AML cells are maintained by the HDAC10-YAP1-PARP1 axis, by blocking this axis may improve the clinical outcomes for FLT3-ITD + AML patients.

We anticipate that this study’s topic will offer readers an intuitive guide to understanding the current status of cutting-edge technologies like PROTACs, which can be employed as tools for targeted therapies and drug development.
